# Notes of an European Tour

**Published:** 1846-09

**Authors:** F. H. Hamilton

**Affiliations:** Rome, Italy


ART. II.—*Notes of an European Tour, by* F. H. HAMILTON, M. D.

ROME, ITALY.

Come now with me along a deserted street, the Via Sacra, leading from the Forum towards the Porta S. Giovanna. We are still within the walls, but without the city — I mean the modern city — one half the space within the gates being occupied by the ancient ruins; and excepting here and there a miserable dwelling with its beggarly occupants, or a solitary convent crowning some one of its seven hills, few are so poor as to live in this deserted, ruinous, miasmatic, ghostly region.

The Fever Hospital of S. Giovanni, is situated in this ancient quarter, not far from the now closed Porta Asinaria, through which both Belisarius and Totila first entered Rome: it is also within a stone's throw of the sacred repository of the "Scala Santa," and occupies a part of the old Papal Palace of Lateran. It can number about 400 beds, and is in all respects the best-ordered and most comfortable hospital in Rome. The beds are neat, and the linen white and clean: in short, every attention requisite for comfort or health is given to the invalids. The patients are chiefly peasants or herdsmen. called *Massari*, brought in from the campagna, or inhabitants of the few miserable dwellings in the neighborhood; for these miasmatic fevers are rare among the dense population of the modern city.

This old Lateran hospital — once or twice destroyed and rebuilt — was for more than a thousand years the Papal residence — from the time of Constantine to the return of the Holy See from Avignon, in 1377; but it was not converted into a hospital until the year 1693. From this same place, therefore, within which now, the "Monks Hospitallers" have become knights of the lancet, and *bleed* and give physic, nine centuries ago did Pope Alexander the 3d send forth his sacred bull in obedience to the decision of the Council of Tours, that since it was the Devil himself who tempted Priests to practise surgery and medicine, as well indeed as *law*, ("now the very respectable Devil Flagel was the soul of the law and the life of the bar,") henceforth none of them should practise either of these wicked, hell-begotten trades, under penalty of excommunication and future damnation. From the same gates also, a half century later, because the iniquitous practise still continued, did Innocent the 3d thunder once more upon the rebellious Priesthood, declaring that, inasmuch as the *Church* abhorred all bloody and sanguinary practises — "Ecclesia abhorret a sanguine" — (Remember the Holy Inquisitiou!) No Priest should be allowed to practise surgery, or perform any operations where steel or *fire* were employed. (Remember the Inquisition!) The sacred benediction and extreme unction shonld also be refused to every poor, lucre-loving brother who did continue hereafter so to practice.

But these Popes are not unlike other men — and with ourselves must be allowed to exercise their own peculiar fancies. Pope Adrian — what *number* I cannot say — kept always the powder of toads about him as a preservative against the plague! and several snccessive Popes have agreed to exclude from their kingdom such heretical works as Richard Mead's Medica Sacra, and the medical writings of Paracelsus, and, for aught I know, a thousand more medical heresies; while Gregory 16th having discovered by the assistance of a very learned Bishop of Lausanne, that mesmerism invoked the aid of Beelzebub, has peremptorily forbidden its practise by the faithful. "Usum magnetismi prout in casu exponitur, non liciter." (Papal Bull of July 1.—1840.)

Nearly opposite the Aventine Hill, on the right bank of the Tiber, is the immense establishment called the 'Hospital of San Michale,' into which are received infirm old people, and also juvenile offenders. I did not visit it.

Near the middle of the city, in the Tiber, is an island dedicated to Esculapius. This island is shaped like a ship with its prow directed against the stream, and in the days of early Rome it contained the temple and sacred altars of the God of Medicine: it is now covered with dense piles of venerable stone buildings, which look as if they might have been the dwelling-houses of such rich old Roman Doctors as Celsus and Asclepiades, the latter of whom had the good luck to be the personal friend and protege of Cicero, whose eloquent praise, I think, must have made his fortune: but Cicero had but a poor opinion of our art generally; and well he might have, for up to his day both medicine and surgery were in a sad state at Rome, and indeed we may say until the birth of Christ, in whose life-time Celsus wrote. During the seven hundred and fifty years preceding which, or from the day of the foundation of Rome, not a single native physician or surgeon had appeared in that city — a fact which must be ascribed to a superstitious abhorrence with which both the plebeians and nobles regarded all the practitioners of medicine and surgery, and which feeling was encouraged by public edicts recommending to the sick religious rites and forms, as the only means of restoration. Patricians also were by statute forbidden to practise either branch of the profession, and both fell necessarily into the hands of slaves, freed-men and priests.

Some opinion may be formed of the state of feeling here at this date, in relation to us, from the remarks of Cato the Censor, in a letter written to his son. "You may depend" says he "upon what I am going to say as a certain prediction. If the Greeks send us their learning, we are undone ; and especially if they send us their *physicians*." In surgery, at least, Cato believed himself superior to all the Greeks, for he was able to reduce a dislocation, he affirms, by chanting the following : "IN ALIO S. F. MOTAS VALTA DARIES, DARDARIES ASTARIES DISSUNAPITUR." If, however, the dislocation was self-willed, and still refused to adjust itself, he had another charm, which was infallible : "HUAT HUAT ISTA PISTA SISTA DOMIABO DAMNAUSTRA"—"ET LUXATO"—"and it is reduced," says the grave Censor. How infinitely indebted ought we to feel to Cato for so valuable a recipe ! for is it not even more simple than the twist-and-pull method of our own great natural bone-setter, Sweet ?—and, withal, so beautifully classic ! I have thought, moreover, that our brethren of the "New School," if they would look diligently, would probably find that Cato was a Homœopathist, and that in this curious collection of words he had forshadowed the advent of that great doctrine, *similia similibus curanter*."—Since the self same words are capable, I have no doubt, of dislocating a jaw, as well as of reducing a hip. Is not the suggestion also rendered yet more probable by the fact that the whole of Cato's treatment was "*vox et praeteria nihil.*"

Of this island and its temple of Esculapius, to which for so long a time the Roman people trusted for relief from their maladies, we gather as follows. 293 years B. C. Rome was scourged with the plague, and the people having consulted the Sibylline oracles, were directed to bring Esculapius from Epidaurus to Rome. On their way home it was ascertained that a serpent had crept into the ship, which they readily believed was the God himself thus transformed. Arrived at Rome, the serpent left the vessel and concealed himself among the reeds of this Island. The plague being soon after stayed, the grateful people walled up the island in the shape of a ship, part of which walls, still remain, and erected upon its centre a massive temple, and transported hither likewise an Egyptian obelisk to serve as a mast. Upon the southern extremity or bow of the ship may still be seen sculptured, and in tolerable preservation, the staff and serpent, emblems of the God. In the church of St. Bartolomeo, which stands upon the site of the ancient altars, are twenty-four granite columns forming the three naives, which were taken from the Esculapian temple. A Hospital also was erected near by, but no remains of it now exist. This must be regarded as one of the earliest of these now numerous and most excellent charities, its foundation dating back more than 2,000 years.

The two bridges which unite this island with the opposite shores of the Tiber, were built, one by Fabricius a street inspector 60 years B. C., as the inscription still shows; the other was erected in the year 467.

Near the northern extremity of the Fabrician bridge is the "Ghetto," or the Jew's quarter ; here, as in every city in Europe, recognized by its narrow streets, crowded population, small dark shops, by its door ways hung out with old clothes, old iron, old crockery, old pictures, old women and old men with foul grey beards. At night and on all public days the gates of their district are closed and locked by order of his Holiness.

La Spirito Santo is on the right bank of the Tiber but higher up, nearly in front of St. Peters church. It is large and richly endowed, but dirty. It contains about one thousand beds. It has six principal physicians and four surgeons, besides numerous assistants, and a large number of medical students who remain constantly in the building, in the capacity of internes and whose board and lodging are given them.

Foundlings and Lunatics have apartments in the same building,

(La Spirito Santo). Among the latter, the straight-jacket system is still in use. The whole establishment is a kind of Bastile, cold, damp and filled with noisome smells; and it does not surprise me that of 12,000 patients received annually, 7 per cent die, and that out of about 4000 foundlings received during five years, about 3000 died! The practice of *swathing* these infants has also, I beleive, some effect in increasing the mortality. They are tied hand, foot, and body in swadling clothes, and in this condition, looking like little mummies bound in linen; they are allowed to lay all day and all night, whether clean or foul is never certain. Here also you may see the old books, and the quaint surgical tools bequeathed by Lancisci the eminent anatomist and surgeon, who died at Rome in 1719. His library once consisting of 20,000 volumes has now mnch diminished. Some of his own writings are there in manuscript.

The Botanic garden is a little below, in the district called the Trastivere, the largest and dirtiest district of Rome. On my way to the garden, I was struck with the peculiar appearance of the inhabitants both male and female. They are the most noble, graceful, intelligent looking peasantry I have ever seen, and I could not but think that if the dirt was washed from their faces, they would constitute the most perfect classic models in the world. They are a people almost distinct from the other Romans, having a dialect and costume peculiar to themselves, claiming also to be the only true descendants of the ancient Romans; and from the close resemblance which they bear to the antique busts of the Vatican, I can hardly doubt the justness of their claim.

The Botanic garden established and supported under the general direction of the Papal university, contains a very clever collection of Medicinal and other rare plants. But I must confess that no plant to me possessed half the interest which attached to the grey and shriven trunk of an "old oak tree" seen in the adjoining grounds of the hermits of St. Jerome. It is all that now remains of Tasso's oak. In this convent the illustrious poet spent the calm evening of his life; and his remains now sleep within the church, underneath a simple marble slab. The oak has long been celebrated in tradition as that under whose broad and refreshing shades Tasso loved most to sit and meditate. It was torn down last year by a violent hurricane, but enough still remains to enable the traveller, to mark the sacrad spot. About a half a mile further on, in the more densely populated part of this district is a very old church standing upon an open square, said by some to be the first ever dedicated to christian worship in Rome. It occupies the site of the ancient "Taberna Meritoria" or hospital established and maintained by the Roman Senate for sick and infirm soldiers. If I am not mistaken, this was the first establishment of the kind, of which history gives us an account. A little farther up the hill of Janiculum, is a church founded by Constantine on the spot where St. Peter is said to have been executed; it is not many rods within the gate of Santa Pancrazio.

Quite in the opposite extremity of the city, near the corso, is the Hospital of Incurables. There is, also, a Hospital for cutaneous diseases, Santa Gallicano, in the Trastevere—a lying-in hospital, Santa Rocco, endowed by a rich cardinal—a hospital for convalescents, Santa Trinita de Pelligrini, near the Monte de Pieta, in addition to the many minor hospitals and infirmaries.

The Pantheon, "Pride of Rome," stands within the modern city, encroached upon on all sides by impertinent buildings which seem combined to crowd, if possible, their venerable and more honored companion from their company. Erected 26 years B. C., it still remains, with its elegant dome, and with its "more than faultless" portico, the admiration and wonder of all nations.

To the left of the entrance, in a niche, now converted into a chapel, lay the remains of Raphael, *skull* and all. That wonderful skull, which possesses an ubiquity equal to that of the true "scala santa," since it may be found both here and in the halls of the academy of St. Luke! But indeed, was not that a most serious joke committed upon the skull of Desiderio de Adjutori, a man without genius or reputation, either in arts or letters, to pass it off as the skull of Raphael? For many years this poor humble skull had remained in the academy the recipient of all the admiration and honor which was due to the "palace" where once dwelt the soul of the great artist. And when the doctrines of Gall were announced, this, with a whole catacomb of other distinguished crania was copied in plaster and sent abroad, to enlighten and convince the skeptical as to the infallibility of Phrenology, by showing plain as the nose upon a man's face, the exact correspondence of every hill and valley of that same skull, with every light and shade of the great artist's character. Mr. Scott, a distinguished Phrenologist, was furnished with a copy, and the readers of the Edinburgh Review were soon delighted with a most minutely detailed drawing of the character and genius of Raphael, which on comparison with the skull, was found to be the exact counterpart of all the cranial indications. The whole world stood astonished at the wonderful simplicity, beauty and perfection of this new science. It was no longer necessary to consult the vague records of history and tradition for dead men's biography, since now with a skull and craniometer "lives and actions" could be so easily "figured" out. Mr. Combe, that he might not be imposed upon by a spurious copy, went himself to Rome, and there saw and handled the identical skull; and so satisfied was he that it answered exactly to all the virtues, vices, affections, talents and other mental endowments of this "unparalleled genius," that in his large work he has given three plates of it, and no less than ten or twelve times referred to it as affording remarkable testimony in favor of his belief. Mr. Gall saw and examined the same skull, and found also the same precise correspondence. But it so happened that in 1833 the tomb of Raphael was opened, in presence of a number of artists, and lo! the whole skeleton lay quietly there, every inch of it, from the sinciput to the calcaneum, measuring in all just 5 feet 7 inches!

I have since seen the copy of Raphael's skull, taken at the time of the disentombment, by Fabris of Rome, and beside it, a copy of the skull of the now dishonored Desiderio, and as to a resemblance in form or size, it does not exist between them.

Of course I have been to St. Peter's church, and counted the "eternal" candles which burn about the tomb of Paul and Peter, and touched the toe of the venerated statue, and walked the ten thousand rooms of the Vatican. I have visited the capitol, and Heaven grant *you* may yet see there the "Dying Gladiator."

——— "His droop'd head sinks gradually low—
And through his side the last drops, ebbing slow
From the red gash, fall heavy, one by one,
Like the first of a thunder shower."

There is nothing which so touches and moves the heart in all Italy—nay I think, in all the world of art.

John Bell, who now lies in the little green cemetry, near the gate of St. Paolo, dwelt enthusiastically upon its anatomical perfection.

And while I speak of statuary, I must remind you that we have, just now, in Rome, a young artist who possesses, I think, most remarkable genius as a sculptor, and is fast gaining laurels both for himself and his country. I speak of Mr. Brown, whose name, I have before mentioned to you. I visited Mr. Brown's studio, close upon the left bank of the Tiber, yesterday, and found him, with his young friend Baker, hard at work. Mr. Brown has the pale thoughtful look of a student, but when he speaks of his art, his cheeks become gradually flushed, and his intelligent black eye sparkles with an uncommoa fire. I have fallen in love with him at first sight, and with scarce a week's acquaintance we knit together like old friends. His Mazeppa, in basso relievo, an original composition, executed in marble in eight days, is a surprising specimen of the rapidity with which he designs and executes. So, also, the elopement of Helen and Pallas. The young Indian boy, is a daring and successful effort. But I am most interested in his new composition, "The David and Goliath," as yet only set in clay. David is represented in the act of carrying the head of Goliath to Saul. The figure is therefore in motion, the left foot being slightly raised, and the left hand being carried back to the left shoulder to draw open the loose robe which in his haste had become displaced. This position of the arm brings into full view the whole breadth of the chest and also enables us to see the beautiful line formed by the body and left leg. In his right hand he carries the hideous and gory head of the giant. Throughout, the figure has a just proportion of lightness for youth, muscularity for strength, and rotundity for such beauty and symmetry as we have always imagined to characterize David. The face is exceedingly handsome, but manly, and is filled with an expression of amiability, zeal and perhaps lofty reliance upon God. In short, I consider it all in all, one of the most anatomically correct and artistically divine creations of genius with which I have yet met.

I am off for Florence, and while I wait for my vetturino, I add as my last mem. for Rome.—The Papal University has five departments. The Department of Medicine has thirteen Professors; Lectures are gratuitous, given in Latin, except cliniques; salaries derived from Papal treasury. Pietro Lupi is Professor of anatomy; Dr. Matthaeis of Medicine; Metax of Zoology and Comparative Anatomy. Charles Lucien Buonaparte, nephew of Napoleon, and Prince of Canino, has a large Zoological collection at his palace, on the Piazza di Vinezia. In Natural History he is ranked as a very eminent scholar, etc. etc.

FLORENCE, TUSCANY.

Remember the card, and when you visit this beautiful "city of the Lily," you will easily find my lodgings. "F. S. Ponson, proprietaire de l'Hotel du Nord a Florence, vis-a-vis la Colonne de S. Trinita: said Colonne was brought many years since from Rome, and has upon its summit a statue of Justice "too high for a Florentine to reach."

I meet here Dr. Syckles, U. S. N., a graduate of our school, also surgeon Wilkins, and some five or six lieutenants, all of whom are out upon furlough, while their vessels are taking in stores at Leghorn. By chance also I fell in at the Cafe Donin, with Drs. Upham of New-York, Parsons of Providence, and Stout of Philadelphia. These with other American ladies and gentlemen, who are now at Florence, and the several resident artistis, form quite a respectable colony, and we have thought some of driving out the natives and taking general possession of the country, under the very puritanic title of New Connecticut. Yesterday standing together on the Ponta St. Trinita, over the classic Arno, we took a vote and we rejoice to announce to you that Tuscany has gone hip and thigh for the whigs!

I made it my first business after having shaken off the dust and fatigue of my journey, to call upon our venerable consul Signor Ambrosi, who is a "fine old Tuscan gentleman," full of wit and good feeling, and who, I have found, submits to a bore with a most martyr-like patience, since although I have thought it necessary to call for his assistance and instruction almost every day I have spent in Florence, yet he has never seemed to weary in his kindness or attention.

Sig. Ambrosi informs me that Sig. Antonio Serantoni the pupil of Mascagni, to whom I had been recommended as the best wax modeler in Italy, is dead, but his successor, Sig. Luigi Calamai is equally skillful. Sig. Calamai is in the constant employ of the Grand Duke, as a wax modeller for his great museum of Natural History, connected with the Pitti Palace. He however is allowed to execute orders, and usually he works under the immediate inspection of C. Burci, Prof. of Pathology, or of Fernandino Zannetti Prof. of Anatomy.

Sig. Calamai has also a small drug shop on the via de cervi, but we found him at his work rooms in the Pitti Palace: from which he accompanied us through the long suit of rooms, I believe numbering twenty, which contain the wax preparations of the museum of Natural History. Lectures are here given every week by able Professors, in the very palace of the Grand Duke, on the several departments of natural science. The wax models, especially those made by Calamai, exceed anything of the kind to be found in Europe, and are only surpassed in number by the famous Alexandrian collection at Vienna. Among them are now many fine anatomical and pathological preparations, both human and comparative. In one room we were shown a representation in wax of a part of the city of Florence, and of the plague as it appeared there in 1348, during which single year 100,000 citizens died. The work is fearfully true to nature, and it was wrought, they say, by Zummo, a Sicilian, under authority of Cosmo the III. The streets and buildings are deserted and reflect the sun from their heated stones with a dry and reddish glare. Upon the scorched pavements lay black and putrid forms, some so bloated that the bowels have burst out, and rats are feeding upon the entrails, while upon the green and yellow face of a dead infant rests a black bug, seeming to sleep there as if already gorged to repletion. Here lies a skull and other fragments of a human skeleton, beside the rotten carcass of a dog — There a mother lately dead, with an infant trying to draw nourishment from the breast— lizards are creeping over them, — a man passing averts his head and covers his nose with a handkerchief. Such are a few of the objects which meet the eye in every direction, all perfect and true — true as death and horrible as the plague. So painfully correct is the work that as we stood in silence, I almost thought I could hear the tinkling of the horse bells, and the hoarse voice of the masked becchini, as they rattled their slow cart from door to door, gathering the dead.

Bocaccio the Poet, who himself was a witness of its ravages in this city, has given us in the introduction to his Decameron, a graphic picture of what he saw. It commenced in the groin and in the axilla as a tumor, which would rapidly attain the size of an apple or an egg; the tumors were called gavoccioli or plague boils. Then similar boils appeared on all parts of the body, and petechiæ occurred in patches on the arms, thighs, &c. Either of these symptoms — the boils or the spots, were deemed fatal. Medicine had no power to alleviate or arrest: a majority died within three days, but others much sooner, and some not quite so soon. "The plague spread itself with the greatest fury, as it ran like fire among dry and oily fuel, and even contact with the clothes and other articles which had been used by the infected, seemed to induce the disease. As it advanced, not only men, but animals fell sick and shortly died, if they had touched things belonging to the diseased or dead. Two hogs were feeding upon the rags of a person who had died of the plague, and after staggering about for a short time, they fell down dead as if they had taken poison."

I will give you M. Calamai's prices for certain anatomical and pathological preparations, in relation to which I made special enquiry. I have also set opposite in brackets M. Guy's prices, as furnished me at his shop in Paris, for the sake of the contrast.

"Memorandum of anatomical wax preparations which Doctor Hamilton has requested of Sig. Luigi Calamai, Professor *de plastique en cire* at the Royal Museum of Florence.

"( The prices as here given are the lowest at which said Professor can furnish these preparations executed in the best manner. The cost of cases and package is not included.)

| | Francs | Francs |
|---|---|---|
| *Eye*, in all its different parts--four times size of life, | 300 | (30) |
| *Ear*, external and internal, exhibiting nerves, arteries, &c. - - - - - - - | 400 | (25) |
| *Head*, with the veins, arteries, and nerves of the face, of the eye and of the ear, - - - - - | 300 | (80 to 160) |
| *Pelvis*, of female, with section of one of the iliac bones, showing all the anatomical parts of this cavity and their connections, - - - - | 450 | (300) |
| *Pelvis*, of male, do do - - - - | 450 | (300) |
| *Genito-urinary* apparatus of male, - - - - | 200 | (30) |
| *Digestive* apparatus complete, (size of life and most perfect, - - - - - - - - | 700 | (300) |
| *Brain*, eight different preparations, (beautiful,) - | 500 | |
| *Great sympathetic nerve*, a complete demonstration in an entire figure, (beautiful,) - - - | 1000 | (600) |
| *Entire figure*, separable to exhibit the different cavities, &c. - - - - - - - - | 3000 | (1500) |
| *Larynx*, showing cordæ vocales, &c. - - | 200 | (18) |
| 10 *Matrices*, showing progress of utero-gestation, &c. - - - - - - - - - - | 800 | (500) |
| *Circulation of Fœtus*, at full term, - - - | 300 | (100) |
| *Venereal diseases*, of face—each prep. - - | 50 to 80 | |
| *Diseases of Eyes*, with face entire—each prep. - | 50 | |
| *Pellagra*, on an arm, - - - - - | 70 | |
| *Phagedenic ulcer*, on an arm, - - - - | 90 | |
| *Diseased Eyes*—each, - - - - - | 15 | (7 to 8) |

The above preparations may all, with a vast many besides, be seen in the museum rooms, and I am bound to say that they exceed any thing I have ever conceived of the perfection of the art of wax modelling—they are life itself, not outdone, but seeming neither more nor less than actual life. For such work his prices are indeed "pas forces"—one half he requires down, and the other half to be paid to the consul when delivered. Yet these prices, it will be seen, are nearly one half higher than the same work can be obtained for at Paris, of M. Guy. Nor is it true, as S. Calamai has said to me, "qu a Paris on ne sait point travailler en cire—les Francais memes le disent." The French certainly do not work as well, and yet their modelling is good, and for class demonstrations it serves the same purpose as the more delicately wrought and more expensive Florentine preparations. The two other "Modelatores in ceri," at present in Florence, are Sig. Calenzoli and Sig. Scrantoni, both inferior to Calamai.

Prof. Burci has charge of the famous physiological and pathological museum in which Mascagni labored,* connected with the large Hospital of Santa Maria Nuova. He here showed me many fine absorbent preparations of his own; also, an arm prepared by Mascagni; in the same room is a bust of this distinguished anatomist and physiologist. Above was a young man dissecting a turtle, whose room and paraphernalia made me feel quite at home. He was exceedingly communicative, and among other things, informed me that there were now attending lectures in Florence about 400 students—that they were allowed 10 subjects per day without cost. Here, also, in the Pathological Museum, I was shown the mosaic table made of *dried* and polished pieces of the different organs and tissues of the human body, prepared by Sig. Segato, who is now dead, having unfortunately carried with him his marvelous secret—for this Sig. Segato claimed, and some have been found sufficiently credulous to believe his statements, that these various specimens, thus arranged, were by him petrified, and then sawed into slabs and polished—and that he had also, in like manner, converted into solid stone, liver, lungs and heart, as well as the entire viscera of the chest and abdomen belonging to frogs, birds and other animals, besides the human body, retaining every where the color of veins, arteries, &c. &c. All of which wonderful things were shown me at my special request, and a greater piece of medical charlatanism was never practised, for excepting some plaster casts of viscera and a few specimens of lung and liver, containing earthy deposites, nothing approaching the consistence of stone can be found in the learned Segato's collection.

* Mascagni was a revolutionist of the Napoleon school, and during a short interval of Austrian success, when a reign of terror prevailed at Florence, second only to that of France, Mascagni was about to be executed for his political opinions, but he was finally saved on the ground of his great worth and literary merit.

The liver is dried up, deformed and black, and as to its *fracture*, it *cuts* like leather. The petrified skin is only parchment, and the petrified centre table is made of similar dried pieces of different viscera cut smoothly, and then varnished; but no where is the natural color preserved except as it is darker or lighter from the varying amount of blood which it contained, or as it is colored by injection. Prof. B. has presented me some of the "petrified" blood, which I shall keep to satisfy the incredulous.

Here stand, also, three entire bodies preserved by injecting them with alcohol, arsenic and vermillion, a method invented some years since by a Dr. Tranchina, of Palermo, in Sicily; the object of which is to keep the body a longer time from decomposition, and at the same time to preserve (by means of the vermillion) the natural complexion. The King of Naples was so delighted with the discovery that he purchased the secret and settled upon him a liberal pension. These three bodies have stood in the museum three years. The skin is not removed, nor are they eviscerated, but preserved entire. The surface is of a redish brown color, and without odour or oil. Prof. B. says the subjects were rather emaciated, and were injected three times in succession from the jugular vein and femoral artery. Dr. Dujat, Demonstrator at the Ecole Pratique, at Paris, recommends the same, and I think I remember also, that it has been used by Dr. Dudley, of Lexington, in the preparation of subjects for dissection, but that it has been found liable to the objectiod that it frequently inflames the fingers of the dissectors. I have myself used, and still should prefer M. Ganal's preparation of salt, alumina and nit. pot. or the creosote and whiskey. Mascagni used a solution of corrosive sublimate.

*Mem.*—The Hospital of S. Maria Nuova is an immense building, situated not far from the great Duomo, and was founded in 1287, by Fulco Portinari, the father of Dante's Beatrice, the "little Bice," she of whom the poet became enamoured, when "clad in that purple vestment," she entertained him so sweetly on the first day of May at her father's house. It contains now about 1040 patients. The wards are very clean—large number of patients laboring under phthisis and scrofula—mercury employed very freely in venereal cases —amputations made by both circular and flap incisions—operations for stone made chiefly by the lithontripter, or by the single or double sub-pubic lateral incisions. The extensive and curious collection of instruments in the surgeon's apartment is well worth an amateur's visit. Prof. B. has given me, nicely done up, a small bottle of male and female tricocephali—wishes me to keep the little wretches as a memento of the Mascagni Museum! I have them snugly corked and sealed, and whether my bottle contains Asmodeus Devil or the Epidaurian serpent I shall learn when I reach home.

The "Ospedale degli Innocenti" and "degli Maternita" is near the church of Annunciation and Prof. Norfini, to whom I was introduced by Sig. Ambrose, is the principal obstetrician. He has twice successfully performed the Cæsarian section. His wards, which I visited in company with himself and Prof. Burci, are well ordered, except that here also I find the "bambino fasciato" — the infant in swaddling clothes, tied tight and half suffocated — and as we pass them lying side and side in their little cribs, they turn up their eyes towards us in a manner most sad and imploring, as if to say, "good Christian pray give us our freedom, for we are in sore bonds." The nurses were evidently attentive and kind to their charges, and one at least was, I make no doubt, intelligent — for she never ceased to talk to us while in her apartment, in a most earnest manner. I think she was reading us a lecture on the virtues of catnip and tansy, or some other precious herb, which, if I could have understood, would have made my fortune for ever among the old ladies at home.

The remaining hospitals are St. Bonifazio, devoted to lunatics, soldiers, cutaneous diseases and incurables — the hospital of St. Giovanni di Dio — of St. Lucia and the Orfanotrofio. Societies for the endowment of poor maidens who get married, exist also in Florence, which bestow annually, it is said, about $15,000!

I remember my promise given at Naples, that I would tell you something more about the society called the Misericordia, when I reached Florence, for it was here that, just 600 years ago, this "compagnia" originated. It was oganized during the Republic, by a large number of noble citizens, who bound themselves mutually to render personal assistance to all who were affected with the plague and were left without friends — to all who were in poverty and fell suddenly sick with any malady — to all who met with severe casualties, without ability to demand assistance, and finally to perform the last Christian services and procure a decent burial to all found dead, and finally, to pay the Priests for their prayers that their miserable souls might soon be released from purgatory. The society has never ceased to exist from that day to this, nor, it is said, has a member ever been known, however occupied, or whatever the personal exposure, to refuse promptly to answer the summons. The city is divided into districts, to each of which a certain number of the fraternity are constantly assigned, and at the signal from the great bell of the Duomo, they immediately draw over themselves a long black robe, with a hood which conceals the face, leaving only the eyes exposed, and hasten to their chapel on the "piazza del Duomo," from whence they are directed to the scene of their duty. When I visited the chapel — a spacious and gloomy hall — I saw several hearses in different parts of the room, with Misericordia in attendance. Upon one of the hearses lay a dead female, with the sacred tapers burning about her, and a Priest performing the last Christian rites previous to burial. To me the scene was novel, and almost painfully solemn and impressive. I have once met a procession of Misericordia consisting of twenty or thirty, carrying upon their shoulders a hearse in which, I understood, was a man recently killed—they were taking him to the burial—and I have occasionally met one or two of these masked and spectre looking men, moving slowly about the streets, presenting to every passer a tin box for alms; and you may distinguish them from every other species of beggar — indeed other beggars are scarcely seen in Tuscany — not only by their strange costume, but by the dignified, gentlemanly, yet almost mandatory air with which they, in silence, present their claim. Take care how you refuse — it may be the Grand Duke himself whose eyes rest so piercingly upon you through those dark port-holes!

